# A PP2A-B56—Centered View on Metaphase-to-Anaphase Transition in Mouse Oocyte Meiosis I

**DOI:** 10.3390/cells9020390

**Published:** 2020-02-07

**Authors:** Leonor Keating, Sandra A. Touati, Katja Wassmann

**Affiliations:** 1 Mammalian Oocyte Meiosis (MOM) UMR7622, Institut de Biologie Paris Seine, Sorbonne Université, 75005 Paris, France; leonor.keating@upmc.fr (L.K.); sandra.touati@upmc.fr (S.A.T.); 2 CNRS UMR7622 Developmental Biology Lab, Sorbonne Université, 75005 Paris, France

**Keywords:** oocyte meiosis, metaphase-to-anaphase transition, spindle assembly checkpoint, error correction, cohesin protection, PP2A

## Abstract

Meiosis is required to reduce to haploid the diploid genome content of a cell, generating gametes—oocytes and sperm—with the correct number of chromosomes. To achieve this goal, two specialized cell divisions without intermediate S-phase are executed in a time-controlled manner. In mammalian female meiosis, these divisions are error-prone. Human oocytes have an exceptionally high error rate that further increases with age, with significant consequences for human fertility. To understand why errors in chromosome segregation occur at such high rates in oocytes, it is essential to understand the molecular players at work controlling these divisions. In this review, we look at the interplay of kinase and phosphatase activities at the transition from metaphase-to-anaphase for correct segregation of chromosomes. We focus on the activity of PP2A-B56, a key phosphatase for anaphase onset in both mitosis and meiosis. We start by introducing multiple roles PP2A-B56 occupies for progression through mitosis, before laying out whether or not the same principles may apply to the first meiotic division in oocytes, and describing the known meiosis-specific roles of PP2A-B56 and discrepancies with mitotic cell cycle regulation.

## 1. Introduction

Any given protein can be modulated by regulating its total concentration, its localization, or its structure and activity. The concentration depends on changes in transcription or degradation, while localization, structure, and activity may be regulated by interaction with other molecules—such as enzymes, inhibitors or activators. Post-translational modifications, typically carried out by enzymes, directly regulate these events.

Protein phosphorylation is among the most common post-translational modifications that regulate signaling inside the cell. They are carried out by kinases: Enzymes that can add a phosphate group to a specific residue on a protein (typically on serine, threonine or tyrosine). These modifications are reversible and can be removed by phosphatases that catalyze their hydrolysis. It is estimated that over 70% of all eukaryotic proteins are phosphorylated [1,2]. These modifications can be constitutive or dynamic throughout the cell cycle (for example, 21% of phosphosites are dynamic during mitotic exit in yeast; [3]). Phosphorylation events occur mostly during cell division in M-phase [4], when condensed chromatin is separated in a highly synchronized and sudden manner to give rise to two daughter cells.

Gametes—sperm and oocytes—are formed through a particular cell division called meiosis. During meiosis, two M-phases occur in sequence without intermediate S-phase to replicate the genome, as would typically happen between two mitotic divisions. Meiotic divisions entail specific cell cycle regulation. Often, the same proteins are necessary both in mitosis and meiosis, but their function must be tweaked for the correct outcome to be achieved. We will review the metaphase-to-anaphase transition during the first meiotic division in mouse oocytes, focusing on the roles of the Protein Phosphatase 2A (PP2A). Particular attention will be given to the roles of PP2A-B56 in correcting errors of kinetochore-microtubule attachment, silencing the Spindle Assembly Checkpoint, and protecting cohesin at the centromeres. Focusing on mammalian oocytes, we aim to put in evidence what remains unclear about PP2A’s function during meiosis.

## 2. Protein Phosphatase 2A—One Name for Multiple Phosphatases

Phosphatases are classified into three super-families by the amino acids they can dephosphorylate: Either serine and threonine, tyrosine, or all three (dual-specificity phosphatases). Most phosphorylation events happen on serine (∼84%), and threonine residues (∼15%), a ratio conserved from yeast to human [3,5,6]. PP2A is a serine/threonine phosphatase, and along with Protein Phosphatase 1 (PP1, another serine/threonine phosphatase), has a pivotal role in regulating M-phase progression.

Serine/threonine phosphatases often form complexes, with several isoforms for each of the subunits. The presence of different isoforms modifies substrate specificity, and this fact compensates for the low number of phosphatases relative to kinases. Concerning PP2A, the PP2A holoenzyme (the complete complex) is composed of a scaffold subunit A, a catalytic subunit C and a regulatory subunit B. With several isoforms for each subunit, PP2A can form several dozen different holoenzymes. Numerous isoforms of the regulatory subunit exist, with B55 and B56 being central for cell division. Additionally, PP2A can exist as a “core enzyme”, composed of scaffold and catalytic subunit alone, but relevant functions of this core enzyme are unknown [7].

Although we decided to focus exclusively on the roles of PP2A-B56 in this review, many other phosphatases are important for progression through mitosis and meiosis, such as other isoforms of PP2A, isoforms of Cdc14 and Cdc25, or PP1, PP4 and PP6. We refer the reader to specialized reviews on the role of those phosphatases during cell division [8,9,10,11,12,13].

### 2.1. Meiosis: Two Sequential Divisions without Genome Replication

Homologous chromosomes, which have previously recombined, are separated into two cells in the first meiotic division, meiosis I. Two sister chromatids per chromosome go into each daughter cell; they are then separated in the second meiotic division, meiosis II, giving rise to haploid gametes with only half of the original genome content. Upon fusion of the two gametes at fertilization, the diploid genome content is re-established.

**Figure 1:**
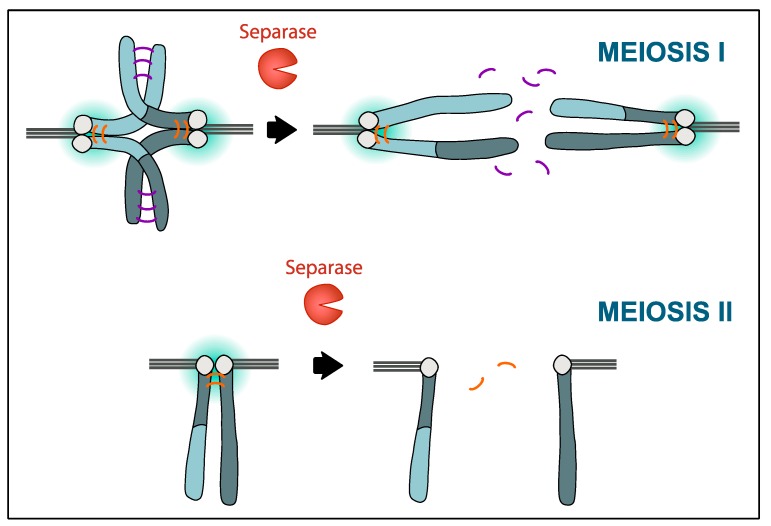
Protection of centromeric cohesin in meiosis I and II. Chromosomes that have recombined (indicated by different color shades), consisting of two sister chromatids each, are held together by cohesin complexes on chromosome arms (indicated in purple) and within the centromere region (orange). Of note, chromosomes in the mouse are telocentric, resulting in the typical cross-shaped appearance of homologous chromosomes in meiosis I. Around the centromere, cohesin is protected by the activity of PP2A-B56 (gradient in turquoise). At the metaphase-to-anaphase transition in meiosis I, Separase is activated and cleaves the cohesin kleisin subunit Rec8 on chromosome arms, to bring about chiasmata resolution and allow segregation of homologous chromosomes to the opposite poles of the spindle. In meiosis II, centromeric cohesin is deprotected at anaphase II onset, allowing Separase to remove the remaining cohesin holding sister chromatids together and hence, their separation. How deprotection of centromeric cohesin is regulated in oocytes to take place only in meiosis II is still unclear.

The two consecutive divisions in meiosis pose challenges that mitotic cells do not encounter, particularly to regulate cell cycle progression and to segregate chromosomes. The first challenge of the cells is exiting meiosis I and entering meiosis II directly, without passing through G1 and S-phase with DNA replication. The second main challenge is to segregate chromosomes correctly. During meiosis I, cells must split homologous chromosomes (held together by their sites of recombination, chiasmata) instead of sister chromatids such as in mitosis. Each pair of sister chromatids must be oriented to the same pole of the meiotic spindle, in a manner never occurring in mitosis. Thus, kinetochore geometry that would typically be detected by the cell as an error in orientation becomes, in meiosis I, the only correct way of separating chromosomes. Once attachments are correct, considering the meiotic context, the protease Separase is activated and cleaves its main substrate, namely, the kleisin subunit of the cohesin complex that keeps chromosomes together. In meiosis, Separase must cleave cohesin in a two-step manner: First along chromosome arms in meiosis I (to allow separation of homologous chromosomes) and then at the centromeres in meiosis II (to allow separation of sister chromatids). This two-step cleavage of cohesin is meiosis-specific and depends on mechanisms that protect centromeric cohesin during meiosis I and remove protection in meiosis II (Figure 1).

### 2.2. Meiosis in Oocytes is Highly Error-Prone

The control mechanisms that ensure correct segregation of the genetic material in mitosis adapt to the specific segregation pattern of meiosis, but seem to lose efficiency, at least in mammalian oocytes. As a consequence, errors in chromosome segregation occur at increased rates. These errors can lead to gametes with an incorrect number of chromosomes, called aneuploid. Aneuploidy in gametes is usually lethal for the developing embryo after fertilization, yet it frequently occurs in mammalian oocytes. Aneuploidy rates in human oocytes are estimated at more than 20%—in stark contrast to the rates of 2–4% seen in sperm—and this percentage increases with maternal age [14,15]. In the last 40 years, we have witnessed an increase in the number of aneuploid pregnancies, as the age of mothers at childbirth increased on average in industrialized countries [16,17].

The duration of female meiosis acutely challenges the oocyte. After premeiotic S-phase in germ cells, oocytes undergo meiotic recombination in the ovary of the developing fetus and are set aside until sexual maturity of the female. Oocytes are arrested in the dicytate stage of meiosis—for up to around 20 months in mice and a few decades in women—until hormonal stimulation at ovulation pushes them to continue the meiotic cycle. Oocytes resume meiosis, undergo the first meiotic division, and start meiosis II. They again arrest at metaphase II and only truly complete meiosis if fertilized, which triggers anaphase II and meiotic exit.

Correct execution of meiosis depends on protein complexes that have to be maintained functional for these long periods of time, as is the case of the cohesin complex, which is not renewed during the extended prophase arrest [18,19,20,21]. Not surprisingly, the correlation between maternal age and error rate results from multiple factors, such as weakening of the cohesin complex, the failure to build a correct meiotic spindle and correctly align chromosomes, and the lower stringency of checkpoint controls [21,22]. To understand why oocytes are so often aneuploid, it is necessary to understand the molecular mechanisms and signaling pathways underlying chromosome segregation in meiosis.

## 3. Stabilization of Kinetochore-Microtubule Attachments

Upon entry into mitosis or meiosis, the nuclear membrane breaks down, a bipolar spindle forms and spindle microtubules attach to chromosomes scattered in the cell, to align them and pull them to the metaphase plate. For this, microtubules from both spindle poles bind to a protein structure called the kinetochore formed at the centromere on chromosomes, and keep pulling. When all kinetochores are correctly attached, the pulling forces from both sides are balanced, and chromosomes are aligned at the metaphase plate. The first attachments to form are often wrong and need time to be corrected. In a process known as error correction, incorrect attachments that do not generate proper tension are detected and destabilized, giving a chance for novel, correct attachments to form [23]. In metaphase of both mitosis and meiosis, a checkpoint, namely, the spindle assembly checkpoint (SAC), recognizes missing attachments at kinetochores and halts the cell in metaphase until all kinetochores are attached. Thereby the SAC is ensuring that anaphase onset does not occur before error correction has been finalized and stable and correct end-on attachments have been established.

Initial attachments between spindle microtubules and chromosomes are formed at random in both mitosis and meiosis and tend to be lateral: kinetochores bind to the side of a microtubule bundle [24]. Lateral interactions of chromosomes with microtubules are mediated by chromokinesins, motors that walk chromosomes towards the plus-end of microtubules and center of the spindle [25,26]. In both budding yeast and mammalian mitosis, Aurora B kinase activity promotes these lateral attachments [27,28]. As chromosomes congress, lateral attachments/interactions gradually become end-on and stable. This transition happens in part because kinetochore proteins have an intrinsically higher affinity towards the extremity of the microtubules than to their sides, making the transition efficient [29].

In meiosis I, as homologous chromosomes segregate, sister kinetochores co-orient and are pulled together to the same pole—the attachment is said to be monopolar. Monopolar attachment in mouse meiosis I is thought to depend on two mechanisms: The kinetochore protein Meikin, that together with Plk1 promotes the close connections between sister kinetochores [30], and chiasmata, which form physical connections between homologous chromosomes at the recombination sites [31]. Monopolar attachments create a particular tension between homologous chromosomes and not sister chromatids, which is meiosis-specific. However, end-on attachments are often merotelic, with the two sister kinetochores binding simultaneously to microtubules emanating from the two spindle poles [32]. In mouse oocytes, each pair of homologous chromosomes undergoes several attempts at biorientation before finally forming stable and tension-bearing attachments. Microtubules pull on chromosomes through these rounds of error correction, translating in a back-and-forth oscillation of chromosomes at the metaphase plate, until they finally stabilize before anaphase [33].

In mouse oocytes, Aurora B/C is required to destabilize attachments for error correction [33,34]. Along with Borealin, Survivin and INCENP, Aurora B/C forms the Chromosomal Passenger Complex (CPC), that phosphorylates multiple substrates at the kinetochore (including Knl1, Hec1, Dsn1, and microtubule depolymerizing kinesins MCAK and Kif2A); and continuously destabilizes microtubule binding [11,35]. If left unchecked, Aurora B/C keeps destabilizing attachments, even if they are correct and under tension [36,37].

PP2A-B56 counterbalances this destabilizing activity of Aurora B/C and ensures correct attachments are left undisturbed until anaphase onset [37]. Hence, establishing correct end-on attachments requires a balance between error correction and PP2A-B56 activity. Interestingly, at least two distinct pools of PP2A localize at the kinetochores of chromosomes in mitosis. Shugoshin (Sgo) recruits PP2A-B56 at the centromere, while the SAC pseudo-kinase BubR1 localizes PP2A-B56 at the outer part of the kinetochore. PP2A recruitment is needed to suppress Aurora B activity and to promote the stabilization of kinetochore-microtubule attachments. Mouse oocytes without PP2A-C (conditional knockout of both alpha and beta isoforms of the catalytic subunit) are unable to form correct attachments, showing that in principle, PP2A is essential for correct attachments also in female meiosis [38]. Therefore, it is tempting to speculate that the localization and/or activity of the distinct PP2A-B56 pools is different in meiosis I compared to mitosis to create a gradient compatible with the specific kinetochore-microtubule geometry of monopolar attached kinetochores. Indeed, like in mitosis, BubR1 is required for establishing robust kinetochore attachments in oocyte meiosis I [39,40]. Surprisingly though, and in contradiction to a hypothesis depending on a role for BubR1-dependent PP2A recruitment for counterbalancing Aurora B activity at kinetochores, BubR1 does not need to be localized to the kinetochore in meiosis I to stabilize attachments [40]. This suggests that if BubR1 stabilizes attachments through PP2A in meiosis, it does not necessarily do so at the kinetochore. Also, chemical inhibition of Aurora B/C in mouse oocytes does not rescue the absence of PP2A, suggesting that PP2A’s role in oocytes goes beyond solely counteracting the activities of Aurora B and C [38]. Additional kinases that PP2A may counteract in oocyte meiosis I for stabilizing attachments, and their respective localization, still need to be identified.

## 4. The Spindle Assembly Checkpoint, PP2A-B56 and PP1

In mitosis, SAC proteins are recruited to unattached kinetochores and generate a soluble inhibitor of anaphase, the MCC (Mitotic Checkpoint Complex). The MCC directly inhibits the Anaphase Promoting Complex or Cyclosome (APC/C) by preventing it from forming an active complex with its co-activator, Cdc20. The APC/C is an E3 ubiquitin ligase; once active, it targets two Separase inhibitors—Securin and Cyclin B1—for degradation. When all kinetochores are correctly attached, the SAC is satisfied. SAC proteins are then removed from kinetochores, and the APC/C-Cdc20 becomes active. Without these inhibitors, Separase becomes free to cleave cohesin on chromosomes and anaphase occurs [41,42,43,44,45]. Once the SAC is satisfied, it stops producing the MCC signal, and several SAC proteins are removed from the kinetochore. In somatic cells, Mps1, the kinase that starts the cascade of SAC protein recruitment, seems to be removed by competition with microtubules when they bind [46,47,48]. Dynein and Spindly strip other SAC proteins (Mad1 and Mad2, among others) away from kinetochores, pulling them along microtubules to the spindle pole [49,50]. However, removing proteins from kinetochores is not enough to inactivate the checkpoint, and the phosphatases PP1 and PP2A are both required for efficient SAC silencing [45,51].

The kinase Mps1 activates the SAC by phosphorylating MELT repeats on the kinetochore scaffold protein Knl1 [45,52]. These modifications recruit all other SAC proteins to the kinetochore in a cascade. However, by phosphorylating these residues, Mps1 also recruits the phosphatase PP2A-B56. PP2A-B56 binds to Knl1 through BubR1, which interacts with the phosphorylated MELTs. Once there, PP2A-B56 can dephosphorylate SILK and RVSF Aurora B motifs on Knl1 to recruit PP1, which, contrary to PP2A-B56, requires a low phosphorylation status of the kinetochore [53,54]. In a feedback loop, PP1 dephosphorylates the MELT repeats on Knl1 to remove SAC proteins and also PP2A-B56. This mechanism silences the SAC, but is also thought to keep the attached kinetochore PP2A-free to quickly re-establish a SAC response in case the attachment is lost. [45,53].

Strikingly though, very recent findings in mitosis indicate that the main function of PP1 and PP2A in silencing the checkpoint is, in fact, to remove Polo-like kinase 1 (Plk1) from kinetochores [55]. Plk1 shares substrate specificity with Mps1, and hence, was proposed to function with the latter in establishing the SAC [56,57,58]. Plk1 binds to Bub1 and BubR1 downstream of Mps1. From there, it phosphorylates MELT motifs that recruit more SAC proteins and amplify the signal, independently of Mps1 [54,55]. Cordeiro and colleagues propose that the main function of PP1 and PP2A-B56 in SAC signaling is to break this positive feedback loop fueled by Plk1 that maintains SAC signaling active, in tissue culture cells. They propose that the main function of PP2A-B56-dependent recruitment of PP1 is not the dephosphorylation of MELT repeats, but to prevent Plk1 from continuing to phosphorylate these sites [55].

## 5. The Spindle Assembly Checkpoint and PP2A-B56 in Oocyte Meiosis I

It is attractive to assume that in oocytes SAC activation and inactivation follow the same rules as in mitosis. In general, this seems to be indeed the case: SAC proteins are recruited to unattached kinetochores in meiosis I (where they are thought to generate an MCC signal as in mitosis), and removed once correct, monopolar attachments are established [22,43,59,60]. However, SAC activity and APC/C inhibition are distinct from mitosis in several aspects.

First, due to their exceptionally large size, oocytes might require local signaling in the vicinity of chromosomes to regulate cell cycle progression and SAC activity, instead of relying on the control of the whole cytoplasmic stock of a given protein. In mouse oocytes, the APC/C ubiquitylates free Cyclin B1 and Securin before targeting the proteins in complexes (Securin with Separase and Cyclin B1 with Cdk1). Also, their degradation happens prior to all kinetochores being perfectly attached and SAC proteins being completely removed [61,62]. This means that some APC/C activity towards Cyclin B1 and Securin is already present in oocytes before the SAC is silenced [63]. Of note, also in mitosis, free Securin has been shown to be preferentially degraded compared to Separase-bound Securin. However, the protection of the bound fraction of Securin depends on the dephosphorylation of Securin by PP2A-B56, that protects it from the APC/C, when bound to Separase [64]. However, the preferential degradation of free Securin and Cyclin B1 in mouse oocytes is regulated in a distinct manner—this activity is independent of SAC control and phosphoregulation [62], and specific to oocytes.

**Figure 2:**
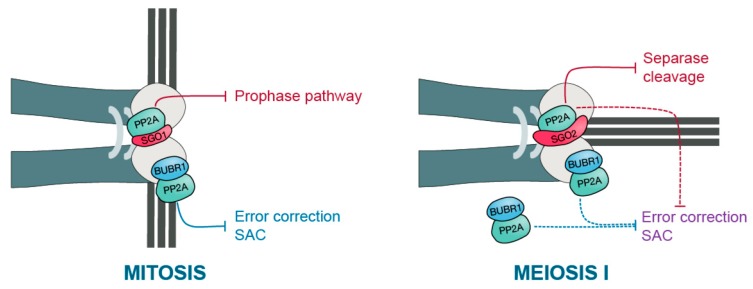
PP2A-B56 kinetochore functions in mitosis and oocyte meiosis I. The scheme depicts the kinetochore region on telocentric murine chromosomes in mitosis (on the left) and meiosis I (on the right). In mitosis, PP2A-B56 is brought to the centromere region by Sgo1 to protect cohesin there from prophase pathway-dependent removal. In parallel, PP2A-B56 is localized to the kinetochore by BubR1 to counteract substrate phosphorylation at the kinetochore, and hence, error correction and SAC activity. In contrast, during meiosis I in oocytes, PP2A-B56 is brought to the centromere region by Sgo2, this time to protect cohesin from Separase-dependent cleavage. Although it also localizes to the kinetochore through BubR1, this BubR1-dependent localization of PP2A-B56 is dispensable for anaphase I onset in oocytes. Either cytoplasmic BubR1-PP2A-B56 or Sgo2-bound PP2A-B56 at the kinetochores may be sufficient to silence error correction and SAC activity in oocytes.

Second, BubR1 kinetochore localization during meiosis does not seem to be required to either establish or silence the SAC. Expressing a BubR1 mutant defective in kinetochore localization in BubR1 knockout oocytes is enough to re-establish the SAC while also allowing its silencing [40]. This indicates that PP2A-B56 does not need to be localized to kinetochores by BubR1 to efficiently turn off the SAC in oocyte metaphase I. Thus, it remains unclear whether the centromeric pool of PP2A recruited by Sgo2 (see below) is able to silence the SAC in oocytes once all kinetochores are attached, or if another phosphatase plays this role in meiosis (Figure 2). We can also speculate that Plk1 phosphorylation of MELT repeats is less efficient in meiosis I, overcoming the requirement for additional PP2A-B56 and PP1 recruitment to silence the SAC.

Third, as in mitosis, in the absence of functional SAC proteins, oocytes activate the APC/C prematurely. However, BubR1-deficient oocytes stay in a metaphase-like state for a prolonged period despite high APC/C activity and low Cdk1 activity [40]. We speculate that PP2A-B56 is recruited through BubR1 for anaphase I progression, and this additional role of BubR1 is revealed in oocytes because PP2A-B56 is not required for SAC silencing in metaphase, such as in mitosis. Other key PP2A substrates may play central roles for either complete APC/C activation, separase activity, or spindle elongation in anaphase I.

## 6. Integrating Error Correction, SAC Control and Anaphase I Onset

Both error correction and SAC control are thought off as a tug-of-war between kinases and phosphatases, with Mps1, Cdk1, Plk1 and Aurora kinases on one side, and PP2A-B56 and PP1 on the other. Kinases start the match unrivaled, but recruit the phosphatases that will outbalance them. The shift from phosphorylation to dephosphorylation is required both to stabilize microtubule attachments and to silence the SAC. How the balance goes from error correction and SAC activity to stable attachments and SAC silencing has been studied extensively, but is still not fully understood.

In mitosis, the change from phosphorylation to dephosphorylation is proposed to happen when, after biorientation, tension across sister kinetochores distances Aurora B from its substrates. Under these conditions, the PP2A-Aurora B balance at the kinetochore would shift to a phosphatase-dominated state [65,66]. This model would explain both SAC silencing and establishment of stable microtubule attachments; yet recent data indicate that the situation is more complex. Several publications show that, both in mitosis and meiosis, the SAC detects attachment status alone and is indifferent to tension or biorientation, suggesting that error correction leads to SAC activation mainly by creating unattached kinetochores [36,45,67]. It is, thus, crucial to turn off error correction once kinetochores are attached, and the SAC is inactivated in metaphase.

Separating Aurora B from its substrates through tension is, therefore, an appealing model to explain how phosphatases stabilize attachments and silence the SAC. However, Knl1 MELT repeats, for example, can still be dephosphorylated and the SAC silenced even under low tension in mammalian cells, when Aurora B would not have been moved away from its substrates [45,68,69]. In mouse oocytes, Aurora B and closely related meiotic Aurora C stay at kinetochores even when tension is applied by the bipolar spindle, but its targets are dephosphorylated [37]. These data indicate that physical, tension-dependent removal of Aurora B is not the only way to prevent phosphorylation of Aurora B substrates.

The Kitajima and Jones labs have proposed that, in mouse oocytes, microtubule stabilization, chromosome biorientation and APC/C activation are independent and happen in a timed manner [37,63]. Yoshida et al. (2015) suggest that the rise in Cdk1 and Plk1 activities as oocytes progress through prometaphase I into metaphase I, sets this timer by recruiting PP2A-B56 [37]. Phosphorylated BubR1 and PP2A-B56 (presumably the BubR1-bound pool) accumulate at the kinetochores during metaphase I and outbalance Aurora B/C, independently of tension. Tweaking their activities by inhibiting Aurora B/C or recruiting more PP2A-B56 (using a BubR1 phosphomimetic mutant) accelerates or delays microtubule stabilization, without affecting chromosome stretching [37]. As a caveat though, it is important to keep in mind that it has not yet been shown that Cdk1 or Plk1 activities indeed set this PP2A timer in oocytes. Lane et al. (2012) propose that the SAC is satisfied as soon as early attachments form and the APC/C is activated several hours before anaphase I [63] further implicating an intrinsic timer of prometaphase I events in oocytes.

Nevertheless, the existence of such a PP2A timer does not prevent activation of the error correction pathway in response to tension-less microtubule attachments in metaphase I. Mouse oocytes do not need to recruit BubR1 to kinetochores for turning off the SAC, such as explained above, yet they still respond to lack of tension in metaphase I. Vallot et al. (2018) show that Aurora B/C phosphorylation of pS55Hec1 increases under low tension, destabilizing attachments [36]. As Aurora B/C tries to correct tensionless attachments, it creates unattached kinetochores that activate the SAC. SAC activity is itself independent of Aurora B/C activity, and it becomes indifferent to tension when Aurora B/C is inhibited. Thus, mouse oocytes progress through meiosis I normally if no major attachment errors are detected. In metaphase I, the absence of sufficient tension is detected in an Aurora B/C kinase dependent manner. The counteracting phosphatase activity is overpowered leading to a delay in anaphase I onset, due to error correction, which generates unattached kinetochores and activates the SAC. It is still unclear how Aurora B/C detects lack of tension, which is especially intriguing in meiosis I, where sister kinetochores are oriented to the same pole and not, as in mitosis, to opposite poles of the bipolar spindle. It is also unknown how error correction is turned off efficiently in metaphase I, and whether BubR1 kinetochore recruitment is needed under these conditions. However, it is attractive to speculate that there are alternative pathways to the tension-dependent physical separation of Aurora B from its substrates to turn off error correction, and those pathways might be dominant in meiosis I.

## 7. Protecting Centromeric Cohesin in Meiosis I but Not Meiosis II

After DNA replication, sister chromatids are held together by cohesin. The cohesin complex is made up of four subunits: Two Structural Maintenance of Chromosomes (Smc1 and Smc3), one kleisin subunit (Scc1/Rad21 in mitosis, and Rec8 and Rad21L in meiosis) and one SA subunit (SA1 or SA2 in mitosis and STAG3 in meiosis). Cohesin forms a protein ring around chromatin, entrapping it. The protease Separase cleaves the kleisin subunit of cohesin to dissociate the complex from chromosomes, releasing the paired sister chromatids. This is a crucial step to segregate the genetic material at anaphase [70,71].

In vertebrate cells, cohesin removal happens in two steps. Most cohesin is first dissociated from chromosome arms by the so-named prophase pathway after being phosphorylated, independently of cleavage. Prophase pathway-dependent cohesin removal requires phosphorylation of the SA2 cohesin subunit and a protein called Wapl. Enough cohesin remains in the centromere region to keep sister chromatids together until anaphase, when active Separase cleaves the remaining cohesin, allowing sister chromatids to separate [72].

During meiosis, cohesin is also removed in two steps, but both depend on cleavage by Separase. This is essential to ensure the specific segregation pattern of meiosis: Homologous chromosomes separate in meiosis I, and sister chromatids in meiosis II. At anaphase I, Separase must cleave cohesin along the chromosome arms (where the two homologous chromosomes have recombined), but spare cohesin holding together the centromere region of sister chromatids [73,74]. There are no indications that prophase pathway-dependent cohesin removal plays a role in addition to Separase for removing cohesin from chromosome arms after dicytate stage, in oocyte meiosis I.

Two proteins play the main role in protecting cohesin at centromeres from Separase cleavage: Shugoshin (Sgo) and PP2A. Sgo is a scaffold protein that interacts with many other proteins at the centromere and kinetochore. Vertebrates present two isoforms of Sgo (Sgo1 and Sgo2), while, for example, budding yeast has just one isoform [75]. In mouse oocytes, the essential isoform is Sgo2, and its presence in the centromere region depends on Mps1 and Bub1 kinases [76]. Sgo2 recruits PP2A-B56, which is thought to dephosphorylate cohesin in the centromere region, thereby keeping it protected from Separase cleavage [73,75]. The interaction between Shugoshin and PP2A is essential for this protection. Budding yeast Sgo1 mutants that fail to bind PP2A are not able to confer protection during meiosis I [77]. In mouse oocytes, interaction between Sgo2 and PP2A is conserved and was shown to be essential for PP2A localization and cohesin protection [78].

Still, several aspects of cohesin protection are unclear. We do not yet know which kinases phosphorylate Rec8 to allow its cleavage in vertebrate oocytes and which sites need to be phosphorylated—or, in fact, if Rec8 needs to be phosphorylated for cleavage at all. In budding and fission yeast, both Casein kinase 1 (CK1) and Cdc7/Dbf4 phosphorylate Rec8 to promote its cleavage by Separase [79,80,81], whereas, in *C. elegans* oocytes, the homolog of Aurora B kinase does the job [82]. In vitro cleavage assays suggest that Plk1 kinase is required to convert mouse Rec8 into a Separase substrate [83], but more recent data indicate that Plk1 is not the main kinase in mouse oocytes (E. Nikalayevich, unpublished results). Independently of mammalian Rec8 being phosphorylated or not, PP2A-B56 localization to the centromere region by Sgo2 seems essential to protect centromeric cohesin and to maintain sister chromatids together throughout meiosis (Figure 2) [78].

Once the oocyte has successfully executed meiosis I—with cohesin at arms removed and homologous chromosomes separated—progression into meiosis II occurs without further delay. There, sister chromatids are separated by removing the remaining cohesin at the centromere. It is still unclear how protection of centromeric cohesin is removed (how centromeric cohesin is “deprotected”) to allow separation of sister chromatids in meiosis II.

Deprotection was first proposed to depend on tension, following the observation that somatic tissue culture cells going through anaphase without tension preserve centromeric cohesin [84]. During meiosis, sister centromeres are held together during meiosis I and are pulled apart for sister chromatid separation in meiosis II. It is, thus, plausible to speculate that deprotection occurs due to tension only in meiosis II, as sister chromatids are pulled apart. Indeed, immunolocalization studies of Sgo2 in male and female meiosis indicated that this model might indeed apply to mouse meiosis [84,85]. However, more recent evidence puts this model in question. Centromere biorientation in budding yeast is not sufficient to render Rec8 cleavable in meiosis II—in this case, deprotection is proposed to depend on the APC/C-Cdc20-mediated degradation of Sgo1 and Mps1 at anaphase II onset [86]. Additionally, mouse and drosophila oocytes seem to depend on a protein counteracting PP2A: SET (also known as I2PP2A) in mouse oocytes [87,88] and NAP1 in Drosophila [89]. Both proteins belong to the same family of Nucleosome Assembly Proteins and are known as histone chaperones [90,91]; SET has also been described as a PP2A inhibitor [92]. SET was shown to colocalize with all three subunits of centromeric PP2A-B56 in meiosis II, but not meiosis I, and importantly, in a tension-independent manner [87]. Although a good candidate for this role, how SET (or NAP1) deprotects cohesin is not clear. For now, there is no formal proof that SET or NAP1 deprotect cohesin by direct inhibition of PP2A, or that the mammalian kleisin subunit of meiotic cohesin complexes has to be phosphorylated in vivo for cleavage by Separase in the first place.

## 8. Protein Phosphatase 2A—One Name for It All

The key to explaining how some substrates are differently phosphorylated in mitosis and meiosis could be in the presence of different pools of PP2A regulatory subunits with distinct phosphatase specificity. Recent developments—of specific PP2A inhibitors and technologies like interactomics and phosphoproteomics—have finally pushed phosphatase specificity into the light, after decades in the dark [93,94]. Our knowledge has leapt forward with the discovery of the PP2A-B56 docking motif, increasing our understanding of how phosphatases recognize their substrates [93,95]. This docking motif—LxxIxE—is essential to localize one of the distinct PP2A-B56 pools to the kinetochore: PP2A-B56 recognizes the LxxIxE sequence on BubR1, thereby increasing the concentration of this phosphatase at this strategic place. Docking motifs also influence the phosphorylation status of the substrates. Mutating the docking site decreases the kinetics of substrate dephosphorylation by modifying the kinase-phosphatase balance [93,96]. As a consequence, disturbing key regulators has a global effect on the timing of cell cycle events. For these reasons, it would be interesting to explore the contribution of LxxIxE motifs on some meiosis-specific proteins, such as those required for cohesion (Rec8), sister kinetochore mono-orientation (Meikin), APC/C regulation (Emi2), or the Cyclin B3 protein that we speculate to regulate APC/C substrate accessibility through phosphorylation [97,98]. All these proteins carry at least one LxxIxE docking motif in their disordered regions. Some of them are likely to play major roles controlling processes specific to meiosis, either by creating a new pool of PP2A-B56 with a particular function or by modulating the phosphorylation of specific substrates.

Unlike for PP2A-B56, no docking motif has been discovered for the other main PP2A regulatory subunit, B55. Only some basic residues upstream and downstream of the phosphosites have been suggested to influence the dephosphorylation rate of substrates [94]. Progressive deletion of these basic residues gradually decreases the affinity of PP2A-B55 for its substrates, leading to a delay in their dephosphorylation. This kind of basic sequence is present frequently in the proteome, which does not allow a systematic description of potential PP2A-B55 targets. However, it is reasonable to expect that a specific PP2A-B55 docking motif will be discovered, significantly increasing our understanding of phosphatase-specificity regulation.

Phosphatase specificity is also influenced by the nature of the amino-acids that its catalytic subunit can recognize. Using phosphoproteomic approaches, it has been suggested that PP2A-B56 and PP2A-B55 present distinct specificity for different kinds of phospho-motifs [94,99,100,101]. The preference of PP2A-B55 for threonine-directed Cdk consensus motifs (p-TP) is conserved from yeast to human and is not observed for PP2A-B56 [94,100]. This result suggests that binding of different regulatory subunits to the catalytic subunit of PP2A could modify its catalytic pocket, leading to distinct substrate specificities.

Phospho-residue specificity is essential to control the timing of cell cycle events in yeast and mammals. In budding yeast, PP2A-B55 (PP2A Cdc55 in *S. cerevisiae*) keeps Cdk phospho-threonines dephosphorylated during interphase. This delays their phosphorylation until Cdk activity reaches a certain threshold allowing the phosphorylation of these substrates. As a consequence, having threonine-directed Cdk sites accessible in the amino-acid sequence of the substrate confers preferential phosphorylation by Cdk only from M-phase on. For example, this is the case for the protein Sli15 (INCENP), a subunit of the CPC complex, and Ndd1, a transcription factor essential for nuclear division and M-phase cyclin expression [99,100].

Another very interesting example comes from the Jacob Nilsson lab, showing that preference of PP2A-B55 for threonine influences the timing of APC/C activation, changing the kinetics of substrate degradation during mitotic exit [102]. The APC/C co-activator Cdc20, which is mostly phosphorylated on threonine residues, is kept in an inactive form by phosphorylation until anaphase onset. After a partial downregulation of PP2A-B55 activity at mitotic entry, PP2A-B55 is reactivated at the metaphase-anaphase transition and can quickly dephosphorylate Cdc20 threonine residues, leading to its activation. In contrast, phospho-serine residues present on the N-terminal of the second APC/C co-activator, Cdh1, delay APC/C-Cdh1 activation. This mechanism contributes to the sequential activation of the two APC/C complexes, which is essential to degrade substrates at the correct time.

We speculate that the same strategy, based on phosphatase specificity, can also order the meiotic cell cycle. The phosphatase preferences might become crucial during the first metaphase-to-anaphase transition when Cdk activity is partially downregulated. After this stage, the second meiotic division takes place without any intermediate S-phase. Some substrates need to be dephosphorylated to restart the chromosome segregation program, while substrates for the DNA replication program have to be kept phosphorylated and inactive to avoid a complete restart of the cell cycle. A new round of DNA replication at this stage would cause aneuploidy in the gamete, and upon fertilization, the generation of an embryo harboring an additional copy of the genome. At the meiosis I to meiosis II transition, PP2A substrate specificity is likely important to dephosphorylate just the key substrates for the execution of the chromosome segregation program only. In budding yeast meiosis, it has already been suggested that the phosphatases’ preference for a phospho-motif favors the dephosphorylation of certain substrates over others [103]. In other organisms, the meiotic cell cycle steps might also be controlled by phosphatases’ preference between threonine and serine phosphoresidues, and their preference for specific phospho-motifs. Future work will aim at determining how phosphatase specificities control progression through the meiotic cell cycle.

## 9. Perspectives

This review is mainly focused on PP2A-B56 because the majority of the mechanisms that we describe for proper chromosome segregation and SAC silencing are under the control of PP2A-B56 in mitosis. It is tempting to speculate that these roles are conserved in meiosis, but we cannot rule out the possibility that some functions attributed to PP2A-B56 are under the control of PP2A-B55 in mouse oocytes. PP2A-B55 plays essential roles to dephosphorylate hundreds of substrates after anaphase onset in mitosis [94], but its specific role and targets during anaphase I in mammals have not been studied extensively. A knock-down of the isoform B55α alpha has been performed in mouse oocytes resulting in a high rate of spindle and chromosome segregation defects. This suggests a crucial role for PP2A-B55 in mouse oocytes [104]. Other studies have used the PP2A inhibitor okadaic acid to study PP2A in mouse oocytes, but unfortunately, this inhibitor does not discriminate the contributions of B55 and B56 in the cell, thereby leaving open the possibility that effects observed have been wrongly attributed to B56 only. The discovery of regulatory-specific PP2A docking motifs extends the possibility of generating specific PP2A-B56 and PP2A-B55 inhibitors, which will help to elucidate the distinct functions of the two holoenzymes in meiosis. To gain insights into how PP2A function is tweaked to give rise to healthy oocytes, it will be essential to understand which pools of PP2A-B56 and PP2A-B55 are active at different stages of meiosis I and to ask where, inside the cell, and towards which substrates, they exert their action overtime.
